# Investigation of Fluid Characteristic and Performance of an Ejector by a Wet Steam Model

**DOI:** 10.3390/e25010085

**Published:** 2022-12-31

**Authors:** Chen Wang, Lei Wang

**Affiliations:** 1 School of Control Science and Engineering, Shandong University, Jinan 250061, China; 2 Department of Mechanical, Aerospace and Civil Engineering, University of Manchester, Manchester M13 9PL, UK

**Keywords:** condensation, supersonic ejector, wet steam model, fluid characteristic

## Abstract

In this paper, a wet steam model is utilized to study the fluid characteristic and performance of a supersonic ejector. The condensation process, which has been ignored by most researchers, is analyzed in detail. It is found that the most intensive condensation happens at the primary nozzle downstream and nozzle exit region. Moreover, the impacts of primary flow pressure and back pressure on ejector performance are studied by the distribution of Mach number inside the ejector. Furthermore, the results show that the secondary mass flow rate first grows sightly then remains almost unchanged, while the primary mass flow rate rises sharply and ejector entrainment ratio drops dramatically with the increase in primary flow pressure.

## 1. Introduction

In recent years, as a passive piece of equipment, the supersonic ejector has gained increasing attention globally within numerous fields, such as fuel cells [1,2], spacesuits [3], refrigeration [4,5], desalination [6], etc.

**Figure 1:**
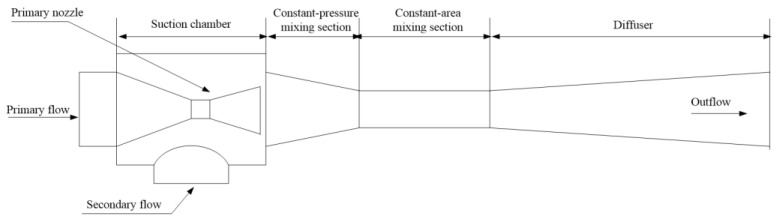
Structure of a supersonic ejector.

The structure of a supersonic ejector is described in Figure 1. Its working principal is as follows: when the primary fluid flows into the ejector nozzle, it is accelerated into a supersonic flow. Then, the secondary fluid is pumped into the suction chamber by a vacuum that the primary fluid created. Next, the two fluids start to mix with the appearance of shock wave and condensation. Finally, the mixed flow enters a diffuser with a subsonic speed.

The ejector is evaluated by entrainment ratio (ER), namely the value of secondary mass flow rate (M_s_) divided by primary mass flow rate (M_p_).

**Figure 2:**
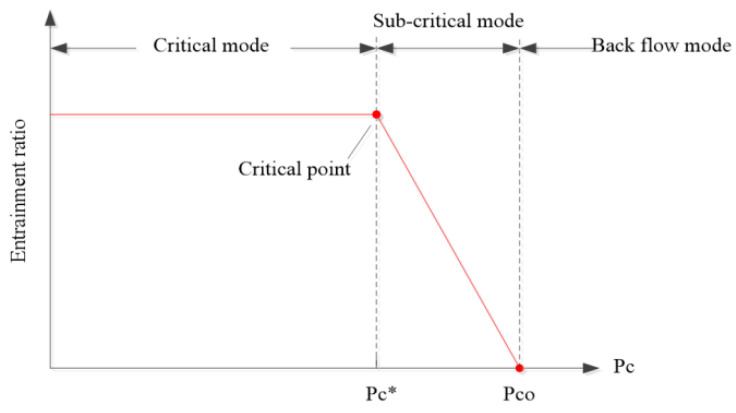
Working modes of the ejector.

It is illustrated in Figure 2 that, with the changing of back pressure, there exist three working modes for the ejector. In the critical model, ER remains in a certain value with the variation of back pressure; however, if the back pressure falls between the critical back pressure (P_c_*) and sub-critical back pressure (P_co_), the ejector begins to operate under the sub-critical mode. ER declines dramatically in according to the rise of back pressure. Then, for the back-flow mode, if the back pressure is higher than P_c_*, ER will be less than zero. There will be a backflow in the ejector, which will make the ejector unable to operate in a normal way. Therefore, it is quite important to ensure the ejector works in the critical mode for a stable and high-efficiency output.

As an initial step, Keenan et al. [7] built the first 1D ejector model for predicting ejector working in the critical mode. Based on the 1D model, Munday et al. [8] proposed the idea of effective area in which the secondary flow is thought to be practically choked when its velocity speeds to a sonic state in the mixing chamber. Then, a shock circle model was designed for the ejector working in the critical mode [9]. Later, an ejector model working in the critical and sub-critical modes was presented by Chen et al. [10]. All the models mentioned above can predict ejector performance with acceptable accuracy; however, they cannot evaluate the influence of ejector geometric parameters, such as nozzle exit position (NXP) and the area ratio (AR), on ejector performance. The impacts of key geometric parameters (NXP, AR, the converging angle of constant-pressure mixing section, the length of constant-area mixing section, etc.) on ejector were evaluated by Yan et al. [11,12,13]; the results show that NXP and AR are the main weighing factor affecting the performance of ejector. When the nozzle exit enters the constant-pressure mixing section, NXP is positive value of the axial distance between nozzle exit and entrance of constant-pressure mixing section, vice versa. NXP = 0 means the axial position of nozzle exit and constant-pressure mixing section entrance coincide. By combing a bellows with the primary nozzle, our research group [14] invented an adaptive nozzle exit position (ANXP) ejector. The NXP of ANXP ejector can be successfully adjusted by the bellows with the change of primary flow pressure, which makes a 35.8% growth of ER under variable working condition.

The condensation phenomenon inside the ejector has been ignored by most researchers. However, condensation, causing numerous tiny droplets, may lead to the erosion of the ejector wall and has a noticeable influence on ejector performance [15,16]. Recently, several numerical investigations have been carried out to study the condensation inside ejector. Zhang et al. [17] optimized the ejector nozzle by a modified wet steam model and found the entrainment ratio predicted by wet steam model is more accurate than that of an ideal gas model. Additionally, compared with an ideal gas model, a wet steam model can predict the condensation phenomena inside the steam ejector. Sun et al. [18] investigated the influence of wall surface roughness and temperature on ejector condensation and performance using a wet steam model. Abadi et al. [19] proposed an in-house code to simulate an ejector with condensation in an unsteady flow. The development of the mixing layer inside an ejector with condensation is studied by Ariafar et al. [20] to explain the reason why the wet steam model can provide a more precise prediction of entrainment ratio than an ideal gas model. Yang et al. [21] simulated the condensation process for an ejector applied in the field of medical injection. Our research group [22,23] investigated the way in which the area ratio, superheated level and surface roughness will impact the condensation inside ejector nozzle using a wet steam model; the results reveal that the three parameters should be cautiously adjusted.

In this paper, a detailed condensation process that occurred throughout the entire ejector is analyzed. Then, the consequence of operation condition including primary pressure and back pressure on the ejector performance and fluid characteristic is investigated. As NXP is a main geometric parameter that has an evident influence on ejector performance, at last, the impact of NXP on entrainment ratio and mass flow rates is evaluated in detail.

## 2. Numerical Modelling

### 2.1. Grid Generation

**Figure 3:**
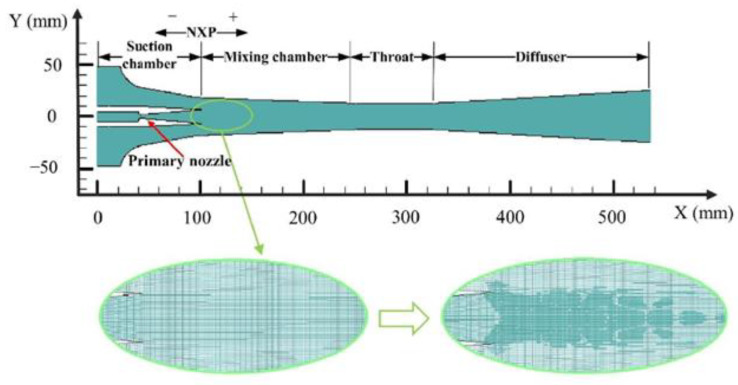
Grid of the ejector used in this study.

The ejector geometry studied in this research is illustrated in Table 1 and Figure 3. The mesh was completed with a quadrilateral grid in Gambit 2.4.6. To predict the ejector supersonic flow with condensation accurately, cells close to the nozzle exit were intensified (shown in Figure 3).

**Figure 4:**
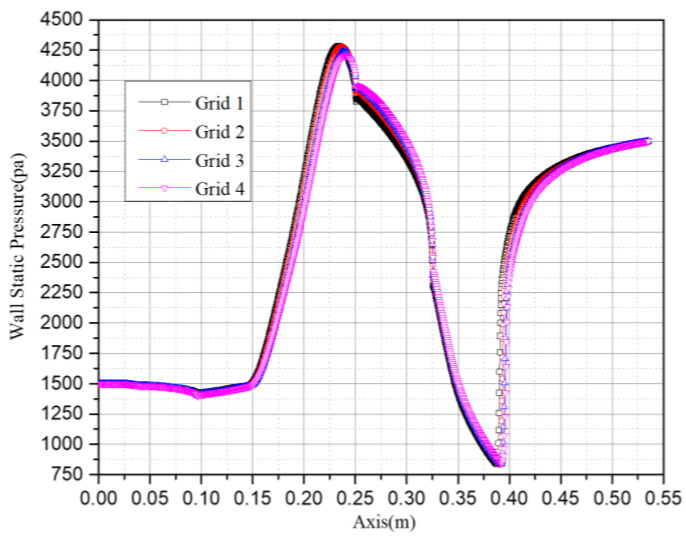
Grid independence test.

The results of the grid impendence test are given in Figure 4. Different levels of grid are compared: grid 1: 229,706 cells, grid 2: 94,525 cells, grid 3: 57,327 cells and grid 4: 41,348 cells. It is shown in the figure that the predicted wall static pressures in the four grids are quite similar; therefore, grid 2 is chosen in the following simulation for its balance between accuracy and computational cost.

### 2.2. Numerical Formulation

#### 2.2.1. Governing Equations

(1) Continuity equation

(1) $$\frac{\partial \left(\rho u_{i}\right)}{\partial x_{i}}=0$$

(2) Momentum equation

(2) $$\frac{\partial \left(\rho u_{j}u_{i}\right)}{\partial x_{j}}=\frac{\partial \tau_{ij}}{\partial x_{j}}-\frac{\partial P}{\partial x_{i}}$$

(3) Energy equation

(3) $$\frac{\partial \left(\rho u_{i}(\rho E+P)\right)}{\partial x_{i}}=\vec{\nabla }\cdot (\alpha_{eff}\frac{\partial T}{\partial x_{i}}+u_{j}(\tau_{ij}))$$

where stress tensor *τ_ij_* is:

(4) $$\tau_{ij}=\mu_{eff}(\frac{\partial u_{i}}{\partial x_{j}}+\frac{\partial u_{j}}{\partial x_{i}})-\frac{2}{3}\mu_{eff}\frac{\partial u_{k}}{\partial x_{k}}\delta_{ij}$$

where *ρ* is density (kg m^−3^), *u* is velocity vector (m s^−1^), *P* is pressure (Pa), *E* is total energy (J), *α_eff_* is effective thermal conductivity (W m^−1^ k^−1^), *T* is static temperature (K), *μ_eff_* is effective dynamic viscosity (N s m^−2^) and *δ_ij_* is Kronecker delta function.

#### 2.2.2. Wet Steam Flow Equations

(1) Mass fraction of droplet

(5) $$\frac{\partial (\rho \beta )}{\partial t}+\nabla \cdot \left(\rho \vec{u}\beta \right)=\Gamma$$

where *β* is the liquid mass fraction and *ρ* is the mixture density defined by:

(6) $$\rho =\frac{\rho_{v}}{\left(1-\beta \right)}$$

$\rho_{v}$ is the vapor density and Γ is the mass generation rate:

(7) $$\Gamma =\frac{4}{3}\pi \rho_{l}Ir_{*}^{3}+4\pi \rho_{l}\eta r_{a}^{2}\frac{\partial r_{a}}{\partial t}$$

where $r_{a}$ is the average droplet radius, $\rho_{l}$ is the condensed liquid density and $r_{*}$ is critical droplet radius:

(8) $$r_{*}=\frac{2\sigma }{\rho_{l}RT\ln Sr}$$

*σ* is the liquid surface tension evaluated at temperature *T*, *Sr* is supersaturation ratio.

(2) The number of the droplets per unit volume

(9) $$\frac{\partial (\rho \eta )}{\partial t}+\nabla \cdot \left(\rho \vec{u}\eta \right)=\rho I$$

which is obtained in the following expression:

(10) $$\eta =\frac{\beta }{\left(1-\beta \right)V_{d}\left(\frac{\rho_{l}}{\rho_{v}}\right)}$$

*V_d_* is the average droplet volume defined as

(11) $$V_{d}=\frac{4}{3}\pi r_{a}^{3}$$

The nucleation rate *I* can be expressed as:

(12) $$I=\frac{q_{c}}{\left(1+\theta \right)}(\frac{\rho_{v}^{2}}{\rho_{l}})\sqrt{\frac{2\sigma }{M_{m}^{3}\pi }}e^{-(\frac{4\pi r_{*}^{2}\sigma }{3K_{b}T})}$$

where *q_c_* is evaporation coefficient, *k_b_* is Boltzmann constant, *M_m_* is mass of one molecule.

*θ* is a nonisothermal correction factor:

(13) $$\theta =\frac{2\left(\gamma -1\right)}{\left(\gamma +1\right)}\left(\frac{h_{lv}}{RT}\right)\left(\frac{h_{lv}}{RT}-0.5\right)$$

where *h_lv_* is the specific enthalpy of evaporation at pressure *p* and *γ* is the ratio of specific heat capacities.

The case is simulated in FLUENT 16.0 and the governing equation combined with the wet steam model is adopted. The working fluid is set as steam. A 2D ejector model is chosen [24] for its advantages of acceptable accuracy and lower computational cost when compared with 3D ejector model. The boundary condition of primary flow and secondary flow is set as pressure inlet and the ejector outlet is set as pressure outlet. Standard k-ε turbulence model with the scalable wall function is chosen as the solver. Second-order upwind scheme approach is utilized for discretizing the convective terms. The SIMPLE algorithm is used for calculating the pressure field. The iteration is assumed as convergent when the residual of all dependent variables is below 1 × 10^−5^.

### 2.3. Model Validation

**Figure 5:**
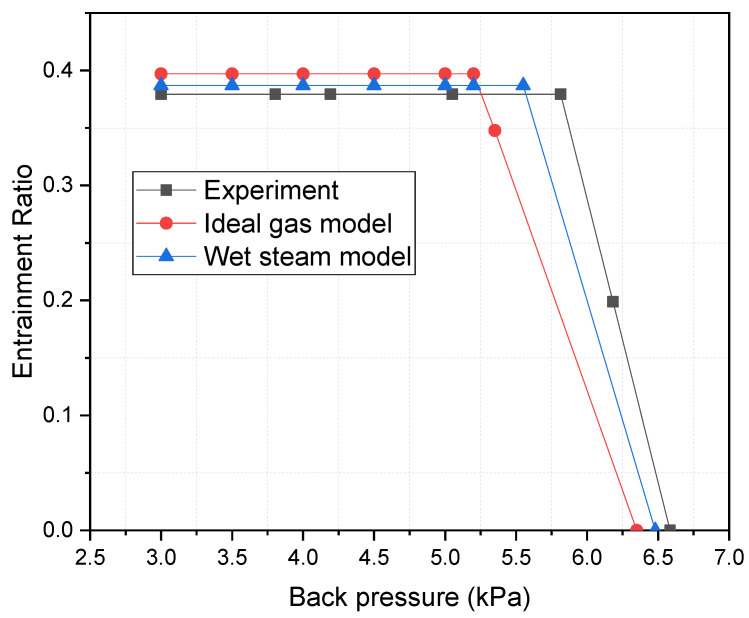
Ejector model validation by experimental data.

The results of ejector validation are illustrated in Figure 5. Both ideal gas model and wet steam model are compared with experimental data [25] under the working condition: primary flow pressure 200 kPa (temperature 120 °C), secondary flow pressure 1.5 kPa (temperature 14 °C) and back pressure ranging from 0 to 6.5 kPa. As shown in the figure, compared with an ideal gas model, the predicted entrainment ratio and critical back pressure by the wet steam model are closer to the experimental data. Therefore, the wet steam model is good enough for the simulation of the ejector.

## 3. Results and Discussion

### 3.1. Condensation Phenomena inside Supersonic Ejector

**Figure 6:**
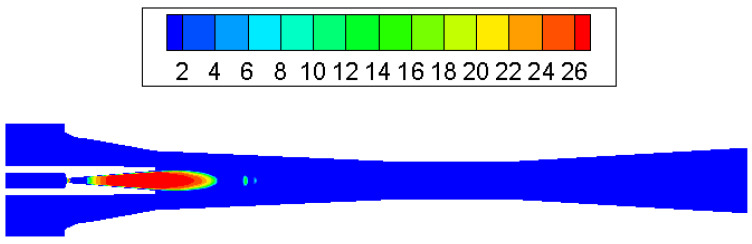
Contour of log10 (droplet nucleation rate) inside ejector.

**Figure 7:**
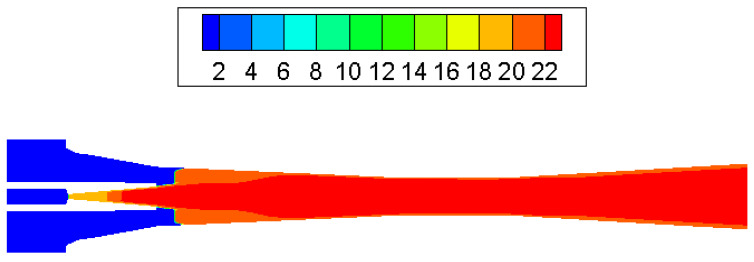
Contour of log10 (droplets per unit volume) inside ejector.

**Figure 8:**
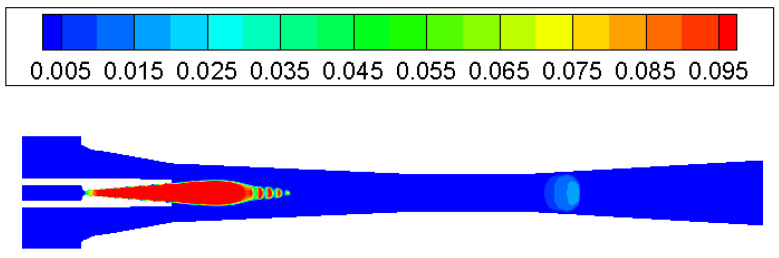
Contour of liquid mass fraction inside ejector.

**Figure 9:**
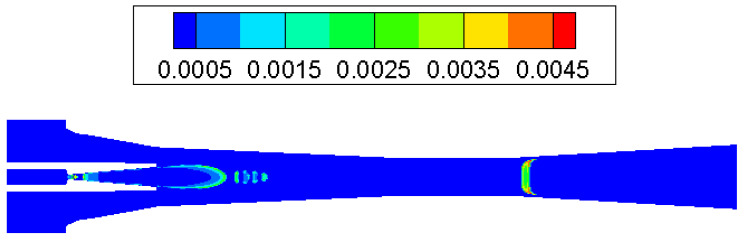
Contour of droplet critical radius (microns) inside ejector.

The condensation process consisted of a nucleation part and a droplet growth part. At the inlet of the ejector nozzle, when the steam temperature declines sharply under rapid expansion, condensation is then likely to happen. Because no other particles exist in the ejector, the nucleation core cannot form immediately. As can be noticed in Figure 6, droplet nucleation rate obtains the most noticeable value in the divergent part and exit region of the ejector nozzle. Then, the vapor condenses rapidly on the nucleation core and droplets start to grow, which will be found in most parts of the ejector (Figure 7). As shown in Figure 8, the liquid mass fraction is mainly located downstream of the ejector nozzle and at the entrance of diffuser. The maximum value of the liquid mass fraction is 0.095 downstream of the ejector nozzle. The droplet critical radius given in Figure 9 further proves the contour of liquid mass fraction, that the critical radius grows bigger further down the diffuser. Therefore, it is concluded that the most intensive condensation is located around the ejector nozzle downstream and the region around the nozzle exit. The second obvious part exists at the diffuser entrance.

### 3.2. The Impact of the Primary Flow on Fluid Characteristic

**Figure 10:**
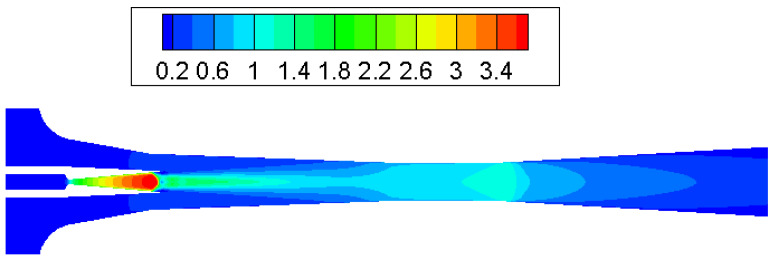
Contour of Mach number in supersonic ejector (P_p_ = 300 kPa, NXP = 6 mm).

**Figure 11:**
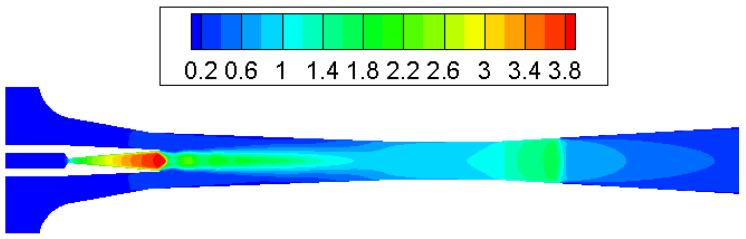
Contour of Mach number in supersonic ejector (P_p_ = 500 kPa, NXP = 6 mm).

**Figure 12:**
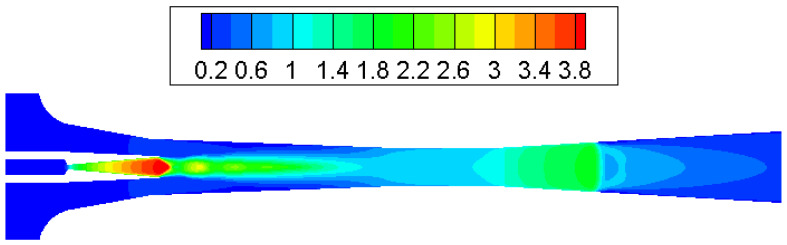
Contour of Mach number in supersonic ejector (P_p_ = 700 kPa, NXP = 6 mm).

**Figure 13:**
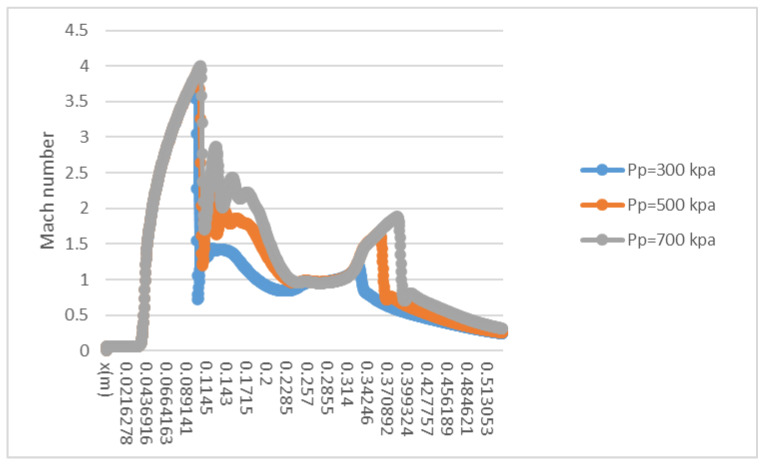
Variation of axial Mach number along the ejector. (NXP = 6 mm).

Contours of Mach number inside the ejector when the primary flow pressure changes from 300 kPa, 500 kPa to 700 kPa are given in Figure 10, Figure 11 and Figure 12. The pressure of the secondary flow and back pressure are fixed at 15 kPa. NXP is fixed at 6 mm in this case. The primary flow speeds to a supersonic state (when Mach number is bigger than one) when it passes through the primary nozzle. Then, the mixing process of two flows is illustrated by the oscillations of velocity along the axis of ejector mixing section (Figure 13). The double shock waves (double-chocking) in the figures indicate that the ejector works normally. The growth of primary flow pressure makes second shock wave in the ejector throat move downward to the diffuser, and the maximum figure of the Mach number in the ejector throat is increased from 1.23 to 1.87.

### 3.3. The Impact of Back Pressure on Fluid Characteristic

**Figure 14:**
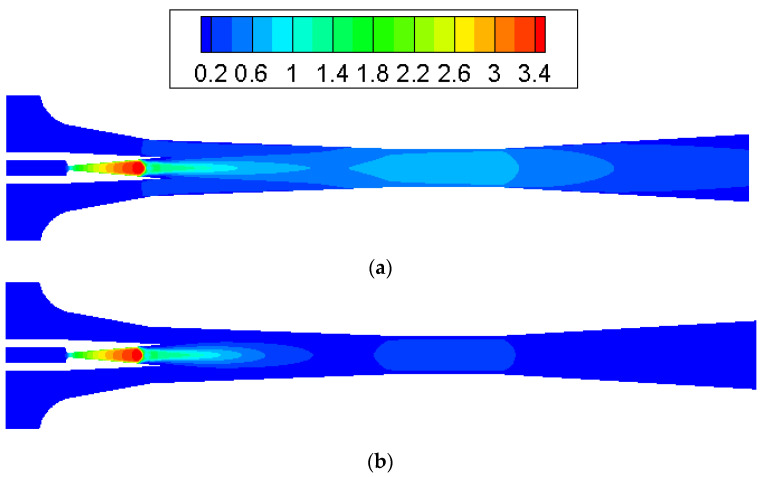

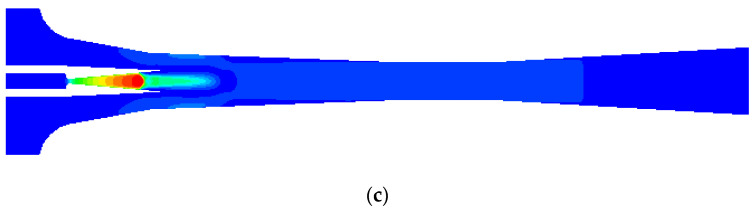
Contours of Mach number inside ejector ((**a**) P_c_ = 15 kPa, (**b**) P_c_ = 16 kPa, (**c**) P_c_ = 17 kPa).

**Figure 15:**
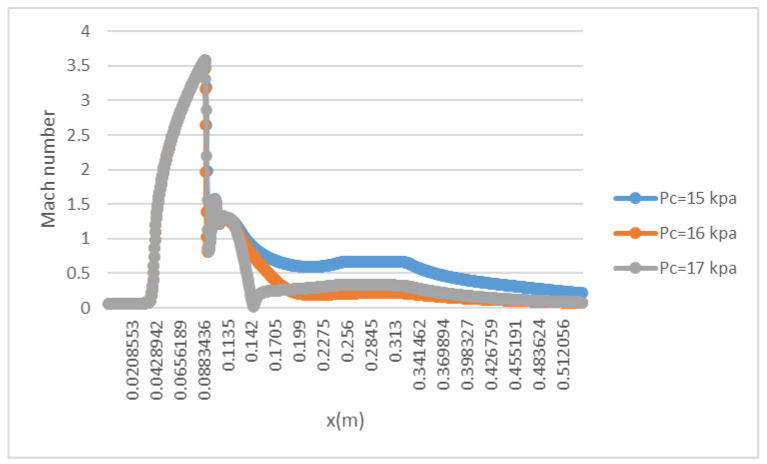
Axial Mach number along the ejector (NXP = 6 mm).

To test the impact of back pressure on the ejector, primary and secondary flow pressures are kept at 200 kPa and 15 kPa, respectively, with NXP = 6 mm. The back pressure is increased from 15 kPa to 17 kPa. Figure 14 illustrates that the critical working mode ejector (Figure 14a) changes to sub-critical model (Figure 14b) and then back-flow mode (Figure 14c). As can be seen in Figure 12, the chock region disappears, which means there is a backflow. This is further illustrated by the axial Mach number of ejector given in Figure 15. Therefore, in this low primary flow pressure condition, the ejector operating mode is quite responsive to a slight change in back pressure. Measures should be taken to make sure the ejector can work normally in this case.

### 3.4. The Impact of Primary Flow and NXP on Supersonic Ejector

**Figure 16:**
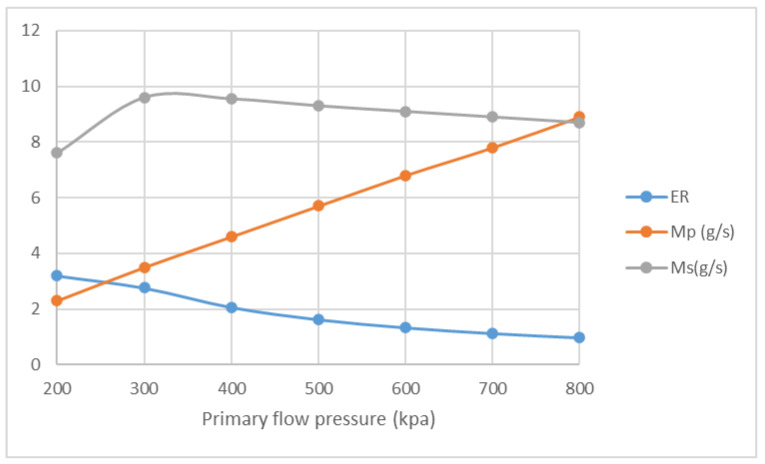
Variation of ER, M_p_ and M_s_ with primary flow pressure (NXP = −30 mm).

**Figure 17:**
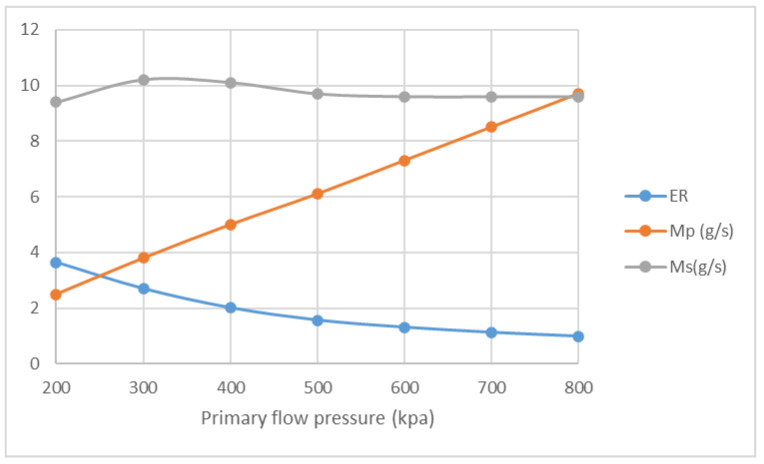
Variation of ER, M_p_ and M_s_ with primary flow pressure (NXP = −6 mm).

**Figure 18:**
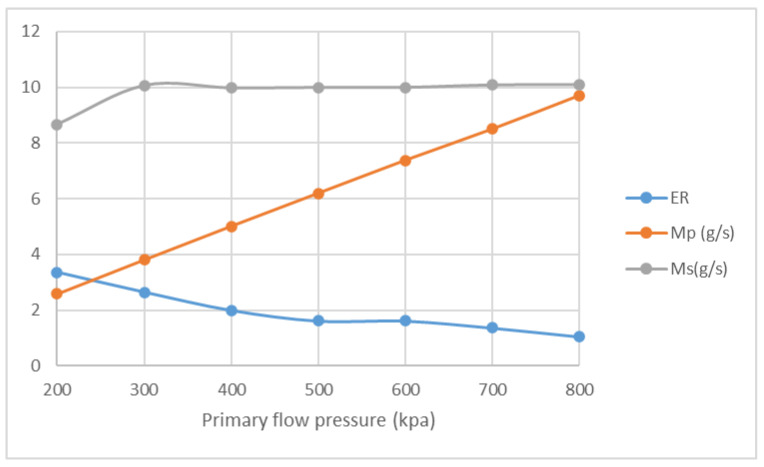
Variation of ER, M_p_ and M_s_ with primary flow pressure (NXP = 6 mm).

**Figure 19:**
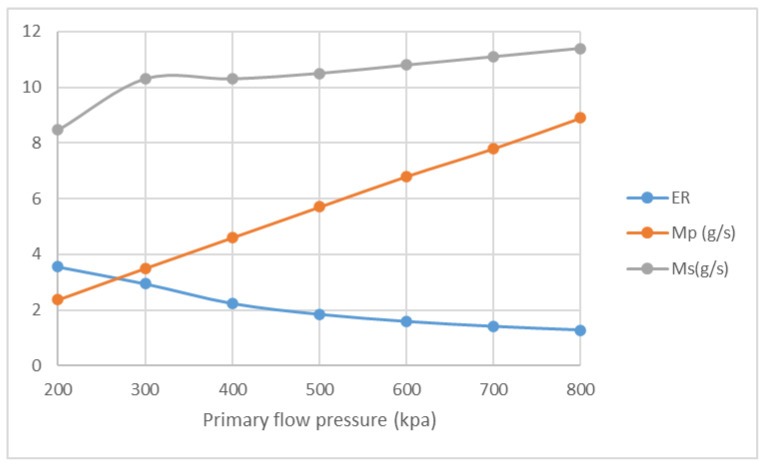
Variation of ER, M_p_ and M_s_ with primary flow pressure (NXP = 30 mm).

The influences of NXP on ER and mass flow rate are studied with the pressure of primary flow ranging from 200 kPa to 800 kPa. The pressure of secondary flow and back pressure are fixed at 15 kPa. NXP is set from −30 mm to 30 mm. The result of Four positions, namely −30 mm, −6 mm, 6 mm and 30 mm, are listed here for comparison. Figure 16 illustrates that, in the case of NXP = −30 mm, ER decreases from 3.19 to 0.98, and M_p_ increases from 2.3 g/s to 8.9 g/s, with the pressure of primary flow growing from 200 kPa to 800 kPa. Both ER and primary flow pressure show similar pattern in Figure 17, Figure 18 and Figure 19. The secondary flow pressure, however, first increases to its peak value of 9.6 g/s at P_p_ = 300 kPa and then declines slowly to 8.7 g/s when P_p_ = 800 kPa (Figure 16). M_s_ first increases sightly and remains almost unchanged around 9.6 g/s, 10.0 g/s for NXP = −6 mm (Figure 17) and 6 mm (Figure 18). The secondary mass flow rate (NXP = 30 mm) increases from 8.5 g/s to 11.4 g/s with the increase in primary flow pressure (Figure 19). The results show that NXP has different influence on the M_s_ with the change in primary flow pressure, and ER declines with the rise in primary flow pressure. Similar works have been performed by other researchers [26,27,28]; through experiment and simulation, they found that NXP has a significant impact on ejector performance. Therefore, it is quite important to choose an appropriate NXP for a specific ejector with its working condition.

## 4. Conclusions

In this paper, the condensation phenomenon that occurred in a supersonic ejector is investigated using a wet steam model. The impact of primary flow pressure, back pressure and NXP on the ejector and its fluid characteristics are then analyzed, and based on and results, the following conclusions can be made:

(1) The most intensive condensation is located around the ejector nozzle downstream, nozzle exit region and the diffuser entrance.
(2) The growth of primary flow pressure will lead to a decline in ER and a downward movement of second chocking position. The ejector may be quite responsive to a tiny change in the back pressure. Measures should be taken to make sure the ejector can work normally.
(3) NXP has different influence on M_s_ with the change of primary flow pressure while ER drops and M_p_ increases sharply based on the rise of primary flow pressure at fixed NXP.

## Figures and Tables

**Table 1:** Key geometry dimensions of the ejector.

Geometry Parameters	mm
Nozzle throat diameter	3.2
Nozzle exit position	6
Mixing chamber length	155
Throat diameter	25.4
Throat length	75
Diffuser length	210
Diffuser exit diameter	50

## Data Availability

Not applicable.
